# Epidemiology of antibiotic-resistant wound infections from six countries in Africa

**DOI:** 10.1136/bmjgh-2017-000475

**Published:** 2018-03-06

**Authors:** Peggy S Lai, Lisa M Bebell, Carron Meney, Linda Valeri, Michelle C White

**Affiliations:** 1 Division of Pulmonary/Critical Care, Massachusetts General Hospital, Boston, Massachusetts, USA; 2 Department of Environmental Health, Harvard T.H. Chan School of Public Health, Boston, Massachusetts, USA; 3 Africa Bureau, Mercy Ships, Cotonou, Benin; 4 Center for Global Health, Massachusetts General Hospital, Boston, Massachusetts, USA; 5 Division of Infectious Diseases, Massachusetts General Hospital, Boston, Massachusetts, USA

**Keywords:** epidemiology, infections, diseases, disorders, injuries, hospital-based study

## Abstract

**Introduction:**

Little is known about the antimicrobial susceptibility of common bacteria responsible for wound infections from many countries in sub-Saharan Africa.

**Methods:**

We performed a retrospective review of microbial isolates collected based on clinical suspicion of wound infection between 2004 and 2016 from Mercy Ships, a non-governmental organisation operating a single mobile surgical unit in Benin, Congo, Liberia, Madagascar, Sierra Leone and Togo. Antimicrobial resistant organisms of interest were defined as methicillin-resistant *Staphylococcus aureus* (MRSA) or *Enterobacteriaceae* resistant to third-generation cephalosporins. Generalised mixed-effects models accounting for repeated isolates in a patient, potential clustering by case mix for each field service, age, gender and country were used to test the hypothesis that rates of antimicrobial resistance differed between countries.

**Results:**

3145 isolates from repeated field services in six countries were reviewed. In univariate analyses, the highest proportion of MRSA was found in Benin (34.6%) and Congo (31.9%), while the lowest proportion was found in Togo (14.3%) and Madagascar (14.5%); country remained a significant predictor in multivariate analyses (P=0.002). In univariate analyses, the highest proportion of third-generation cephalosporin-resistant *Enterobacteriaceae* was found in Benin (35.8%) and lowest in Togo (14.3%) and Madagascar (16.3%). Country remained a significant predictor for antimicrobial-resistant isolates in multivariate analyses (P=0.009).

**Conclusion:**

A significant proportion of isolates from wound cultures were resistant to first-line antimicrobials in each country. Though antimicrobial resistance isolates were not verified in a reference laboratory and these data may not be representative of all regions of the countries studied, differences in the proportion of antimicrobial-resistant isolates and resistance profiles between countries suggest site-specific surveillance should be a priority and local antimicrobial resistance profiles should be used to guide empiric antibiotic selection.

**Key questions**

**What is already known about this topic?**

- Little is known about the antimicrobial susceptibility of common bacteria responsible for wound infections in sub-Saharan Africa.

**What are the new findings?**

- Here we report antimicrobial susceptibility data on bacterial isolates obtained from patients clinically suspected of having wound infections.
- Cultures were collected from Mercy Ships, a non-governmental organisation that operated a single mobile surgical unit in Benin, Congo, Liberia, Madagascar, Sierra Leone and Togo between 2004 and 2016.
- In multivariate analyses, we found the proportion of isolates with antimicrobial resistance differed significantly between these countries, including methicillin-resistant *Staphylococcus aureus* and *Enterobacteriaceae* resistant to third-generation cephalosporins.

**How might this influence practice?**

- We provide antimicrobial resistance profiles for isolates obtained from patients with suspected wound infection who live in countries where previously little to no antimicrobial surveillance data are available to guide empiric treatment.
- Though antimicrobial resistance isolates were not verified in a reference laboratory and these data may not be representative of all regions of the countries studied, our finding that resistance profiles differ by country suggests the need for ongoing surveillance for antimicrobial resistance and development of local antibiograms.
- Empiric antibiotic choices should be guided by use of local antimicrobial resistance profiles.

## Introduction

Resistance of microbes to commonly available antibiotics, or antimicrobial resistance, is a growing global public health crisis.^1^ A report commissioned by the Wellcome Trust and UK Department of Health estimated that by 2050, 10 million deaths annually worldwide will be attributed to antimicrobial resistance.^2^ The global burden of infectious disease is disproportionately concentrated in sub-Saharan Africa,^3^ yet there remains a lack of comprehensive antimicrobial resistance data from this setting. A recent WHO global report on surveillance of antimicrobial resistance lacked information from the majority of the countries in sub-Saharan African countries or lacked data on priority pathogens such as methicillin-resistant *Staphylococcus aureus* (MRSA).^4^ Appropriate treatment of infections in this region is hampered by poor access to and the prohibitive cost of diagnostic tests to identify pathogens and their antimicrobial susceptibility patterns, the dearth of adequately trained laboratory personnel, lack of microbiology laboratory infrastructure, inadequate consumables required for diagnostic testing or prescriber under-appreciation of the value of laboratory data to guide antimicrobial choice and duration.^5^ Current treatment guidelines in sub-Saharan Africa focus on empiric treatment of infections as there is often no local antimicrobial susceptibility data. However, this strategy can lead to adverse patient outcomes. For example, based on limited available antimicrobial resistance data, Zaidi *et al*^6^ reported that 70% of neonatal bloodstream infections in resource-limited settings would not be adequately treated by the WHO-recommended empiric regimen of ampicillin and gentamicin. In these settings, there is a conundrum. On the one hand, inappropriate use of broad-spectrum antimicrobials drives antimicrobial resistance. On the other hand, in many resource-limited settings, lack of access to effective antimicrobials kill more people than antimicrobial resistance.^1^ These observations demonstrate the crucial need for antimicrobial susceptibility data to guide antimicrobial therapy in sub-Saharan African countries.

Mercy Ships is a non-governmental organisation that operates a self-contained mobile surgical unit equipped with a microbiology laboratory on a hospital ship, providing free surgical care in sub-Saharan Africa. Each year, this floating hospital visits a different host country, with patients presenting from urban, periurban and rural areas of the host country. Mercy Ships works closely with local governments and hospitals to reach potential patients throughout each host country. For each field service, patient screening and selection, surgeries, inpatient and outpatient postoperative care occur over a 10-month period in that country, after which the hospital and associated facilities are disinfected and shut down, and the ship returns to a shipyard for maintenance and repairs. A new field service then typically starts in a different country the following year. This organisational structure provides a unique opportunity to obtain antimicrobial susceptibility data from a number of resource-limited countries in sub-Saharan Africa without the added variability introduced by laboratory practices, personnel and equipment.

The primary aim of this study was to provide important descriptive data on rates of antimicrobial resistance from wound infections obtained during field services in the following six sub-Saharan African countries that have previously lacked or had very little published antimicrobial susceptibility data: Benin, Congo, Liberia, Madagascar, Sierra Leone and Togo. A secondary aim was to test the hypothesis that the proportion of antimicrobial-resistant clinical isolates differed between countries.

## Methods

A retrospective review of microbiological culture data was collected from field services performed by Mercy Ships during surgical outreaches in Benin, the Republic of Congo, Liberia, Madagascar, Sierra Leone and Togo between 2004 and 2016. Mercy Ships is staffed by mostly western-trained volunteers and offers free elective surgeries in the following specialties: general, plastics and reconstruction, orthopaedic, maxillofacial, ophthalmic and obstetric fistula surgeries. Each field service operates for 10 months after which all patients are discharged, facilities such as hospital wards and ancillary services (including the microbiological lab) are disinfected and shut down, and the ship returns to a central shipyard (typically the Canary Islands) for repairs and maintenance. At the beginning of each field service, the operative procedures offered by Mercy Ships are advertised through (1) radio, newspaper and public announcements in all districts of each country; (2) notification of all health facilities and (3) word of mouth. Patients present to Mercy Ships from urban, periurban and rural areas, and eligibility screening of patients occurs either during *en masse* screenings at the beginning of each outreach, smaller screenings throughout the outreach or referrals from local health facilities. Eligible patients are given an appointment to return prior to their operative date, where a physician examines them in the off-ship admissions department, performs preoperative testing and admits them to the hospital ward located on the ship. Surgeries and in-patient postoperative care is provided on board the floating hospital. After hospital discharge, outpatient nursing wound care or physical therapy treatment is provided as required for up to 10 months.

The floating hospital is equipped with a comprehensive microbiology lab staffed by three full-time laboratory technologists, one of whom is a senior laboratory scientist charged with supervising the daily operations of the lab and staff. Standard operating protocols and bench aids are developed and updated by the Laboratory Programme Manager for Mercy Ships, an off-site position. Laboratory technologists are certified by the American Society of Clinical Pathology or a peer organisation, and laboratory policies closely mirror Clinical Laboratory Improvement Amendments (CLIA) standards. The microbiology lab participates in routine quality control procedures using internal quality control indicators for microbiological growth and antimicrobial susceptibility testing. However, due to the limited supply of media and difficulty obtaining stock organisms in-country, quality control using reference strains is not routinely performed.

Wound swabs for microbiological culture are collected when ordered by the treating physician based on clinical concerns for a wound infection. Typical criteria for wound infection include warmth, swelling, pain or drainage from the surgical site, or the presence of fever or unexplained leucocytosis. Wounds are first cleaned by wiping away surface exudate with sterile saline or 70% alcohol. A sterile swab is passed deep into the base of the wound to firmly sample the fresh border. Wound culture samples may be collected preoperatively, intraoperatively or postoperatively, transported at room temperature to the microbiology laboratory and inoculated onto sterile culture media in the order of least selective to most selective medium. Choice of inoculation media is described in standard operating protocols and depends on culture source. Agar types used for wound cultures include MacConkey, Tryptic Soy Agar (TSA) 5% Sheep Blood and Chocolate, where appropriate. Samples inoculated onto TSA 5% Sheep Blood and Chocolate agar are then placed at 36°C in a CO_2_ GasPak for 24 hours for anaerobic culture. Samples inoculated onto MacConkey agar are placed at 36°C for 24 hours. Gram stains are also prepared from each sample. Growth on media is quantified after 24 hours, and anaerobic cultures are reincubated for an additional 24 hours if no growth is seen. Aerobic cultures are kept at 4°C for 7 days prior to disposal. When growth is seen, subculture is performed on each distinct colony type. Simultaneous growth of three or more organisms is reported as ‘mixed’ growth of bacteria.

Microbial identity and antimicrobial susceptibility testing are performed using the Siemens Microscan system (Neg Breakpoint Combo Panel 41 and Pos Combo Panel Type 21) according to the manufacturer’s instructions, including using a negative control sample for growth. In brief, three isolated colonies from a freshly cultured agar plate are selected from the agar plate and suspended within 4 hours of MicroScan testing. Oxidase testing is performed on all Gram-negative organisms prior to MicroScan testing. Antimicrobial susceptibility breakpoints are determined using the Minimum Inhibitory Concentration (MIC) of antimicrobial agents and based on the most recent Clinical and Laboratory Standards Institute (CLSI) guide released at the time susceptibility determinations are made. Microbial identity and antimicrobial susceptibility are reported on a standardised form and given to the treating physician, who chooses the antibiotic they considered most appropriate given the clinical and microbiological information available. Clinical data, including patient medical record number, age, gender, date and site of specimen collection, organism identified and antimicrobial susceptibility patterns are recorded in an unstructured format into a central database.

For this study, wound culture data were extracted from the central database and re-entered into a structured format using WHONET software V.5.6.^7^ The primary outcome of interest was the detection of antimicrobial-resistant bacteria. This was defined as microbiological detection of MRSA or *Enterobacteriaceae* resistant to third-generation cephalosporins. Routine swabs screening for MRSA carriage were not included in the analysis.

Summary statistics are described by mean±SD for continuous variables or number (percentage) for categorical variables. Univariate testing was performed using analysis of variance for continuous variables and Fisher exact test for categorical variables. To test the hypothesis that proportions of bacteria with antimicrobial resistance differed by country, we used generalised mixed-effects models. A six-level categorical variable for country (with Madagascar as the reference group) was used as the primary predictor. Covariates include patient gender and age, wound location (abdomen/pelvis, chest/breast/back, groin/genitalia, head and neck, lower extremity and upper extremity) and hospital location where the culture was obtained (preadmission, inpatient, operating room and outpatient locations). Subject-specific random intercepts were included to account for repeated isolates obtained from the same participant, with subjects clustered within field service to account for potential correlation within field service due to case mix. For the primary outcome of MRSA, the analysis was performed only on *S. aureus* isolates (n=856). For the outcome of *Enterobacteriaceae* resistant to third-generation cephalosporins, the analysis was performed only on *Enterobacteriaceae* isolates (n=662). All statistical analyses were performed in R 3.3.3. Generalised mixed-effects models were implemented using the *MASS* package.^8^ A two sided P value <0.05 was considered statistically significant.

## Results

Antimicrobial susceptibility data from the following field services were collected: Benin 2004 and 2016; Congo (also known as the Republic of Congo, or Congo-Brazzaville) 2013, Liberia 2005, 2007 and 2008; Madagascar 2014 and 2015; Sierra Leone 2011; and Togo 2010 and 2012. In total, 3145 isolates from 1262 patients were collected, with median of 2 (IQR 1–3) isolates per patient. Table 1 shows summary statistics regarding the isolates. The average age of patients was 24.6±15.7 years and 55.8% were males. The majority of the isolates (59.6%) were collected in the inpatient setting, followed by the outpatient (25.4%), operating room (8.0%) and preadmission settings (7.0%). Overall, 27.2% of isolates were identified as *S. aureus*, 21.0% of isolates as *Enterobacteriaceae*, 12.5% as *Pseudomonas* spp, 5.8% as *Streptococcus* spp, 4.4% as *Acinetobacter* spp and 1.1% as *Enterococcus* spp.

**Table 1:** Characteristics of patient isolates from wound cultures stratified by country

	Overall	Benin	Congo	Liberia	Madagascar	Sierra Leone	Togo	P value
Number of isolates	3145	687	461	440	654	466	437	
Patient characteristics								
Age in years	24.6±15.7	26.1±15.2	23.0±13.6	25.3±15.7	22.1±15.0	28.5±18.4	22.5±15.1	<0.001
Male gender	1728 (55.8)	430 (63.0)	203 (46.7)	257 (58.7)	302 (46.7)	311 (66.9)	225 (52.2)	<0.001
Ward								<0.001
Preadmission*	205 (7.0)	96 (14.2)	29 (6.3)	11 (2.6)	43 (6.8)	11 (3.6)	15 (3.5)	
Inpatient	1745 (59.6)	297 (43.9)	282 (61.2)	281 (66.4)	307 (48.3)	251 (82.6)	327 (76.6)	
Operating room	233 (8.0)	44 (6.5)	29 (6.3)	30 (7.1)	64 (10.1)	28 (9.2)	38 (8.9)	
Outpatient	744 (25.4)	240 (35.5)	121 (26.2)	101 (23.9)	221 (34.8)	14 (4.6)	47 (11.0)	
Wound location								<0.001
Abdomen/pelvis	149 (4.7)	28 (4.1)	7 (1.5)	55 (12.5)	31 (4.7)	12 (2.6)	16 (3.7)	
Chest/breast/back	106 (3.4)	40 (5.8)	15 (3.3)	8 (1.8)	16 (2.4)	16 (3.4)	11 (2.5)	
Groin/genitalia	261 (8.3)	47 (6.8)	12 (2.6)	11 (2.5)	51 (7.8)	120 (25.8)	20 (4.6)	
Head and neck	1127 (35.8)	287 (41.8)	183 (39.7)	118 (26.8)	259 (39.6)	123 (26.4)	157 (35.9)	
Lower extremity	903 (28.7)	201 (29.3)	142 (30.8)	143 (32.5)	180 (27.5)	122 (26.2)	115 (26.3)	
Upper extremity	19 (0.6)	81 (11.8)	102 (22.1)	93 (21.1)	116 (17.7)	71 (15.2)	117 (26.8)	
Unclassified†	580 (18.4)	3 (0.4)	0 (0.0)	12 (2.7)	1 (0.2)	2 (0.4)	1 (0.2)	
Organisms identified								
*Staphylococcus aureus*	856 (27.2)	191 (27.8)	113 (24.5)	116 (26.4)	172 (26.3)	166 (35.6)	98 (22.4)	<0.001
*Enterococcus* spp	26 (1.1)	4 (0.8)	0 (0.0)	4 (1.2)	8 (1.7)	4 (1.1)	6 (1.9)	0.220
*Streptococcus* spp	133 (5.8)	22 (4.4)	13 (4.1)	12 (3.7)	59 (12.4)	18 (4.9)	9 (2.8)	<0.001
*Pseudomonas* spp	287 (12.5)	63 (12.7)	52 (16.5)	42 (13.0)	41 (8.6)	30 (8.1)	59 (18.5)	<0.001
*Acinetobacter* spp	102 (4.4)	26 (5.2)	15 (4.8)	14 (4.3)	22 (4.6)	17 (4.6)	8 (2.5)	0.595
*Enterobacteriaceae*‡	662 (21.0)	162 (23.6)	98 (21.3)	87 (19.8)	141 (21.6)	83 (17.8)	91 (20.8)	0.292
*Proteus mirabilis*	187 (5.9)	31 (4.5)	31 (6.7)	24 (5.5)	38 (5.8)	24 (5.2)	39 (8.9)	
*Klebsiella pneumoniae*	139 (4.4)	39 (5.7)	20 (4.3)	8 (1.8)	42 (6.4)	16 (3.4)	14 (3.2)	
*Escherichia coli*	99 (3.1)	37 (5.4)	8 (1.7)	11 (2.5)	22 (3.4)	10 (2.2)	11 (2.5)	
*Enterobacter cloacae*	75 (2.4)	28 (4.1)	13 (2.8)	11 (2.5)	12 (1.8)	4 (0.9)	7 (1.6)	
*Morganella morganii*	37 (1.2)	8 (1.2)	4 (0.9)	11 (2.5)	5 (0.8)	7 (1.5)	2 (0.5)	
*Providencia stuartii*	22 (0.7)	2 (0.3)	3 (0.7)	2 (0.5)	4 (0.6)	4 (0.9)	7 (1.6)	
*Citrobacter diversus*	20 (0.6)	7 (1.0)	2 (0.4)	2 (0.5)	2 (0.3)	4 (0.9)	3 (0.7)	
*Serratia marcescens*	14 (0.4)	2 (0.3)	2 (0.4)	5 (1.1)	4 (0.6)	1 (0.2)	0 (0.0)	
*Enterobacter aerogenes*	9 (0.3)	2 (0.3)	3 (0.7)	1 (0.2)	1 (0.2)	1 (0.2)	1 (0.2)	
*Citrobacter freundii*	9 (0.3)	1 (0.1)	2 (0.4)	0 (0.0)	5 (0.8)	0 (0.0)	1 (0.2)	

Presented as mean±SD or number (%).

P values from analysis of variance for continuous variables or Fisher’s exact test for categorical variables.

*Preadmission locations include sites of patient screening to determine eligibility for surgeries offered by Mercy Ships, admissions tent located off the ship where patients undergo routine health examination including blood draw and microbiological testing as determined necessary by the admission physician (typically a general practitioner), or dental clinics located off ship where patients present for same day procedures.

†Recorded culture data lacked sufficient information to classify site of wound.

‡Ten most commonly isolated *Enterobacteriaceae* species listed below.

Detailed antimicrobial resistance profiles are as listed in table 2 for selected Gram-positive organisms. Overall, a significant proportion of *S. aureus* isolates were resistant to oxacillin (23.9%), though there was significant variability between countries, being highest in Benin (34.6%) and Congo (31.9%), and lowest in Madagascar (14.5%) and Togo (14.3%). Resistance of *Staphylococcus aureus* isolates to other antimicrobials was common, including to ciprofloxacin (18.9%), trimethoprim/sulfamethoxazole (55.7%) and clindamycin (8.8%). Although there were few isolates of *Enterococcus* spp, no vancomycin resistance was detected.

**Table 2:** Gram-positive isolates resistant or intermediate-susceptibility to tested antimicrobials, overall and stratified by country

	Overall	Benin	Congo	Liberia	Madagascar	Sierra Leone	Togo	P value
*Staphylococcus aureus*	856	191	113	116	172	166	98	
Oxacillin	205 (23.9)	66 (34.6)	36 (31.9)	24 (20.7)	25 (14.5)	40 (24.1)	14 (14.3)	<0.001
Gentamicin	89 (10.4)	29 (15.2)	30 (26.5)	7 (6.0)	8 (4.7)	9 (5.4)	6 (6.1)	<0.001
Ciprofloxacin	162 (18.9)	40 (20.9)	26 (23.0)	6 (5.2)	49 (28.5)	26 (15.7)	15 (15.3)	<0.001
TMP/SMX	477 (55.7)	110 (57.6)	64 (56.6)	26 (22.4)	109 (63.4)	113 (68.1)	55 (56.1)	<0.001
Clindamycin	75 (8.8)	3 (1.6)	15 (13.3)	9 (7.8)	22 (12.8)	11 (6.6)	15 (15.3)	<0.001
*Enterococcus* spp	26	4	0	4	8	4	6	
Ampicillin	8 (30.8)	0 (0.0)	0 (0.0)	2 (50.0)	3 (37.5)	2 (50.0)	1 (16.7)	0.420
Gentamicin	22 (84.6)	4 (100.0)	0 (0.0)	1 (25.0)	7 (87.5)	4 (100.0)	6 (100.0)	0.009
Ciprofloxacin	10 (38.5)	1 (25.0)	0 (0.0)	1 (25.0)	4 (50.0)	2 (50.0)	2 (33.3)	0.852
Erythromycin	17 (65.4)	4 (100.0)	0 (0.0)	2 (50.0)	6 (75.0)	2 (50.0)	3 (50.0)	0.419
Vancomycin	0 (0.0)	0 (0.0)	0 (0.0)	0 (0.0)	0 (0.0)	0 (0.0)	0 (0.0)	–

Presented as number (%).

P values from the Fisher exact test for categorical variables.

TMP/SMX, trimethoprim/sulfamethoxazole.

Detailed antimicrobial resistance profiles are as listed in table 3 for selected Gram-negative organisms. A large proportion (24.5%) of *Enterobacteriaceae* isolates were resistant to ceftriaxone, a third-generation cephalosporin. Again, there was significant variability between countries, being highest in Benin (35.8%) and lowest in Madagascar (16.3%) and Togo (14.3%). Resistance to other antimicrobials was common in *Enterobacteriaceae* isolates, with overall 21.3% resistant to fluoroquinolones and 22.1% resistant to both ampicillin and gentamicin.

**Table 3:** Gram-negative isolates resistance or intermediate-susceptibility to tested antimicrobials

	Overall	Benin	Congo	Liberia	Madagascar	Sierra Leone	Togo	P value
*Enterobacteriaceae*	662	162	98	87	141	83	91	
Ampicillin	496 (74.9)	146 (90.1)	83 (84.7)	35 (40.2)	100 (70.9)	65 (78.3)	67 (73.6)	<0.001
Gentamicin	172 (26.0)	50 (30.9)	26 (26.5)	33 (37.9)	26 (18.4)	20 (24.1)	17 (18.7)	0.009
Ceftriaxone	162 (24.5)	58 (35.8)	25 (25.5)	24 (27.6)	23 (16.3)	19 (22.9)	13 (14.3)	0.001
Ciprofloxacin	141 (21.3)	47 (29.0)	25 (25.5)	22 (25.3)	20 (14.2)	17 (20.5)	10 (11.0)	0.003
TMP/SMX	406 (61.3)	111 (68.5)	57 (58.2)	62 (71.3)	77 (54.6)	52 (62.7)	47 (51.6)	0.016
Imipenem	15 (2.3)	1 (0.6)	1 (1.0)	2 (2.3)	1 (0.7)	4 (4.8)	6 (6.6)	0.013
Amp+Gent	146 (22.1)	50 (30.9)	26 (26.5)	11 (12.6)	25 (17.7)	19 (22.9)	15 (16.5)	0.006
Quin+Ceph3	102 (15.4)	41 (25.3)	16 (16.3)	14 (16.1)	12 (8.5)	11 (13.3)	8 (8.8)	0.001
Resistant to ≥2 classes*	159 (24.0)	54 (33.3)	21 (21.4)	27 (31.0)	24 (17.0)	21 (25.3)	12 (13.2)	0.001
Resistant to ≥3 classes*	85 (12.8)	32 (19.8)	15 (15.3)	11 (12.6)	10 (7.1)	8 (9.6)	9 (9.9)	0.023
Resistant to ≥4 classes*	2 (0.3)	0 (0.0)	0 (0.0)	1 (1.1)	0 (0.0)	0 (0.0)	1 (1.1)	0.363
*Pseudomonas* spp	287	63	52	42	41	30	59	
Gentamicin	67 (23.3)	10 (15.9)	10 (19.2)	11 (26.2)	15 (36.6)	4 (13.3)	17 (28.8)	0.097
Ciprofloxacin	48 (16.7)	11 (17.5)	6 (11.5)	7 (16.7)	9 (22.0)	4 (13.3)	11 (18.6)	0.816
Imipenem	19 (6.6)	1 (1.6)	4 (7.7)	3 (7.1)	3 (7.3)	2 (6.7)	6 (10.2)	0.559
*Acinetobacter* spp	102	26	15	14	22	17	8	
Gentamicin	26 (25.5)	8 (30.8)	5 (33.3)	5 (35.7)	4 (18.2)	1 (5.9)	3 (37.5)	0.278
Ciprofloxacin	27 (26.5)	10 (38.5)	3 (20.0)	2 (14.3)	2 (9.1)	7 (41.2)	3 (37.5)	0.105
TMP/SMX	60 (58.8)	14 (53.8)	8 (53.3)	10 (71.4)	12 (54.5)	11 (64.7)	5 (62.5)	0.873
Imipenem	2 (2.0)	0 (0.0)	0 (0.0)	0 (0.0)	2 (9.1)	0 (0.0)	0 (0.0)	0.191

Presented as number (%).

P values from the Fisher exact test.

*Antimicrobial classes defined as third-generation cephalosporins, aminoglycosides, fluoroquinolones and carbapenems.

Amp+Gent, Ampicillin + Gentamicin; Quin+Ceph3, Quinolone + third-generation cephalosporin; TMP/SMX, trimethoprim/sulfamethoxazole.

In multivariate testing (table 4), country remained a significant predictor of isolating MRSA (P=0.002). Using Madagascar as the reference country, *S. aureus* isolates from Congo and Benin had significantly higher odds of being methicillin-resistant (OR 5.36 (2.06–13.95), P<0.001 for Benin vs Madagascar; OR 5.05 (1.68–15.16), P=0.004 for Congo vs Madagascar). Similarly, country remained a significant predictor of *Enterobacteriaceae* resistant to ceftriaxone (P=0.009). *Enterobacteriaceae* had higher odds of being resistant to third-generation cephalosporins in Benin and Congo as compared with Madagascar (OR 9.76 (2.62–36.40), P=0.001 for Benin vs Madagascar; OR 6.11 (1.44–25.88), P=0.015 for Congo vs Madagascar).

**Table 4:** Association between country of patient origin and isolation of antimicrobial-resistant bacteria

	OR (95% CI)	P value
MRSA		
Togo vs Madagascar	0.41 (0.09 to 1.93)	0.258
Sierra Leone vs Madagascar	1.93 (0.49 to 7.57)	0.349
Liberia vs Madagascar	2.25 (0.40 to 12.53)	0.355
Congo vs Madagascar	5.05 (1.68 to 15.16)	0.004
Benin vs Madagascar	5.36 (2.06 to 13.95)	0.001
*Enterobacteriaceae* resistant to third-generation cephalosporins		
Togo vs Madagascar	2.38 (0.39 to 14.39)	0.345
Sierra Leone vs Madagascar	2.48 (0.39 to 15.87)	0.338
Liberia vs Madagascar	6.46 (0.67 to 62.34)	0.108
Congo vs Madagascar	6.11 (1.44 to 25.88)	0.015
Benin vs Madagascar	9.76 (2.62 to 36.40)	0.001

Generalised mixed-effects model used to adjust for repeated isolates in a patient, potential clustering by field service, age and gender of patient, wound location and hospital location where wound culture obtained. Country is a significant predictor of methicillin resistance among *Staphylococcus aureus* (overall P=0.002) and resistance to third-generation cephalosporins among *Enterobacteriaceae* isolates (overall P=0.009).

MRSA, methicillin-resistant *Staphylococcus aureus*.

## Discussion

We present antimicrobial resistance data on key pathogens from clinical wound isolates of patients presenting to a single floating hospital ship from six African countries with a paucity of pre-existing data. We found a high proportion of isolates resistant to commonly used first-line antimicrobials. There are, however, significant differences in antimicrobial resistance between these countries in both unadjusted and adjusted analyses, even between geographic neighbours, suggesting that antimicrobial susceptibility profiles from one sub-Saharan African country cannot be a substitute for another.

Literature on antimicrobial resistance in the countries studied here has been extremely sparse.^4 9^ Prior reviews have suggested that antimicrobial resistance is increasing over time and significant enough to impact clinical care in resource limited settings.^10^ Here, we report that the majority of *Enterobacteriaceae* isolates in the population we sampled are resistant to ampicillin, and a substantial proportion are resistant to gentamicin, often first-line antibiotics recommended for surgical site infections in healthcare facilities of the countries studied.^11 12^ We also found a high proportion of the isolates resistant to fluoroquinolones and third-generation cephalosporins, antibiotics commonly used throughout sub-Saharan Africa. Lastly, 23.9% of *S. aureus* isolates were methicillin-resistant, a concerning finding for resource-limited settings where alternative antibiotics such as vancomycin are not routinely available. Our results highlight the crucial need to gather more antimicrobial resistance data from countries in sub-Saharan Africa in order to guide empiric treatment choices and to determine which antimicrobials should be available in these settings.

The proportion of *Enterobacteriaceae* and *S. aureus* resistant to antimicrobials differed between countries studied. However, the mechanisms driving between-country differences are not clear. We were not able to incorporate local prescribing practices, availability of antimicrobials, prior antimicrobial use in participants and related factors in our analysis, as this information was not available retrospectively. However, the relatively low proportion of antimicrobial-resistant isolates seen in Madagascar and Togo in this analysis does provide hope that high rates of antimicrobial resistance are not universal.

A major strength of our study is that we report on a large number of isolates sampled from patients presenting from multiple sub-Saharan African countries where prior antimicrobial resistance data are sparse or non-existent. This is a single-centre study, thus there is likely less variability in patient characteristics between countries (based on protocolised patient screening and selection procedures and the limited types of surgical procedures that are offered), surgical staff, sample handling and processing protocols, and laboratory personnel expertise than if this was a multicentre study conducted in six different countries. Our study does have some limitations. The focus of Mercy Ships is on surgical patients and our isolates were from wound cultures; different antimicrobial resistance patterns may be found in other patient populations and sample types. Nosocomial infections can affect resistance rates, and we presented all isolates from patients rather than choosing one isolate per patient, thus the generalisability of our data on antibiotic resistance to other healthcare settings in the countries studied has to be interpreted in this context. Our study was retrospective and we were therefore unable to perform genetic testing for mechanisms of antimicrobial resistance. Also, the antimicrobial resistance patterns of highly resistant isolates, such as carbapenem-resistant *Enterobacteriaceae*, were not confirmed in a reference laboratory due to monetary and logistical constraints. In most countries studied, no reference laboratory exists, and sending potentially infectious human samples internationally for verification was not feasible. In addition, though Mercy Ships expends considerable effort to recruit patients from throughout the host country by working with the Ministry of Health, in-country hospitals and non-governmental organisations for screening and referral of patients, our data may not be representative of all regions of the countries studied. To ensure access to Mercy Ships resources reached as far as possible, significant resources were used to recruit patients living far from the port where the floating hospital is docked,^13^ including screening patients across the host country and providing free transportation. Lastly, year and country of field service were confounded, and despite performing analyses adjusted for year of sample collection, it was difficult to assess whether rates of antimicrobial resistance increased over time. Despite these limitations, we present a large amount of antimicrobial resistance data on common pathogens that is likely to be clinically useful when interpreted in context, and will help address a large gap in knowledge of antimicrobial resistance data from sub-Saharan Africa.

In summary, we found that resistance to locally available antimicrobials was common among wound infection isolates and differed significantly between the six countries included in this analysis. Future studies should investigate why some of these sub-Saharan African countries, such as Madagascar and Togo, have significantly lower rates of antimicrobial resistance than other countries such as Benin and Congo. This may provide clues to effective policies that can prevent worsening of this important public health problem. More surveillance data on antimicrobial resistance from sub-Saharan Africa are needed to guide local treatment of infectious diseases.
